# An automated ICU agitation monitoring system for video streaming using deep learning classification

**DOI:** 10.1186/s12911-024-02479-2

**Published:** 2024-03-18

**Authors:** Pei-Yu Dai, Yu-Cheng Wu, Ruey-Kai Sheu, Chieh-Liang Wu, Shu-Fang Liu, Pei-Yi Lin, Wei-Lin Cheng, Guan-Yin Lin, Huang-Chien Chung, Lun-Chi Chen

**Affiliations:** 1 Department of Computer Science, Tunghai University, Taichung, Taiwan; 2 Department of Critical Care Medicine, Taichung Veterans General Hospital, Taichung, Taiwan; 3 Department of post-Baccalaureate Medicine, College of Medicine, National Chung Hsing University, Taichung, Taiwan; 4 Supervisor of Nursing Department, Taichung Veterans General Hospital, Taichung, Taiwan; 5 Department of Nursing, Taichung Veterans General Hospital, Taichung, Taiwan; 6 Department of Industrial Engineering and Enterprise Information, Tunghai University, Taichung, Taiwan; 7 College of Engineering, Tunghai University, Taichung, Taiwan

**Keywords:** Motion detection, Deep learning, Video streaming data, ICU, RASS

## Abstract

**Objective:**

To address the challenge of assessing sedation status in critically ill patients in the intensive care unit (ICU), we aimed to develop a non-contact automatic classifier of agitation using artificial intelligence and deep learning.

**Methods:**

We collected the video recordings of ICU patients and cut them into 30-second (30-s) and 2-second (2-s) segments. All of the segments were annotated with the status of agitation as “Attention” and “Non-attention”. After transforming the video segments into movement quantification, we constructed the models of agitation classifiers with Threshold, Random Forest, and LSTM and evaluated their performances.

**Results:**

The video recording segmentation yielded 427 30-s and 6405 2-s segments from 61 patients for model construction. The LSTM model achieved remarkable accuracy (ACC 0.92, AUC 0.91), outperforming other methods.

**Conclusion:**

Our study proposes an advanced monitoring system combining LSTM and image processing to ensure mild patient sedation in ICU care. LSTM proves to be the optimal choice for accurate monitoring. Future efforts should prioritize expanding data collection and enhancing system integration for practical application.

**Supplementary Information:**

The online version contains supplementary material available at 10.1186/s12911-024-02479-2.

## Introduction

Assessing sedation in non-communicative critically ill patients is crucial. Excessive sedation can prolong mechanical ventilation and increase morbidity and mortality, while insufficient sedation may cause agitation, anxiety, and pain [1, 2]. Hence, an evaluating sedation tool is crucial for monitoring the sedation levels of critically ill patients. Sedation tools, such as the Bispectral index (BIS) [3], are recommended but not universally available. Currently, nurse-protocolized (N-P) targeted sedation protocols, employing scales like the Richmond Agitation-Sedation Scale (RASS), are commonly used [4–6]. Unfortunately, they cannot continuously monitor the sedation levels to titrate the sedatives.

The COVID-19 pandemic has prompted a heightened emphasis on wireless sensing technologies to reduce human interactions and prioritize non-contact healthcare, particularly for healthcare workers, to mitigate virus spread [7]. Utilizing continuous and remote non-contact monitoring systems has proven effective in detecting various health conditions such as sleep disorders, heart failure, arrhythmia, activity levels, and stress [8–11]. This approach aligns with infection control measures and enables real-time optimization of care through fine-tuned treatment strategies.

Recent advances in deep learning and AI models have gained popularity in clinical applications for disease diagnosis and prevention. Fang et al. developed a video-based non-invasive respiration monitoring system that detects infants’ respiratory frequency to alert caregivers to potential incidents and mitigate Sudden Infant Death Syndrome (SIDS) risks [12]. Another study demonstrated the effectiveness of deep learning-based pain classifiers using facial expressions for automated pain assessment in critically ill patients, achieving promising accuracy in both image- and video-based classifiers. Additionally, deep learning can be applied to screen for depression, observe behaviors, track posture, and monitor epilepsy [8, 13].

Our study aimed to design an AI-assisted automatic classifier of agitation, which could be applied in a non-contact, continuous sedation monitoring system. The system could aid nurses in assessing and monitoring the movement of intensive care unit patients and facilitate timely intervention and treatment based on the assessment outcomes. Using artificial intelligence and deep learning, we successfully extracted the features of real-time video and constructed the models to classify the agitation status automatically.

## Materials & methods

### Setting

This study was conducted in the intensive care units of Taichung Veterans General Hospital (TCVGH), a 1530-bed medical center in central Taiwan. The study was approved by the Institutional Review Board and Ethics Committee of TCVGH (IRB No. CG21307B). Informed consents were obtained, and digital video recordings of ICU patients were taken without disrupting standard care. Exclusion criteria were applied to patients under 20, pregnant individuals, and HIV patients. The patient’s age, sex, RASS, and restraint status were recorded too.

### Research framework

#### The study consisted of seven major steps

**Fig. 1:**
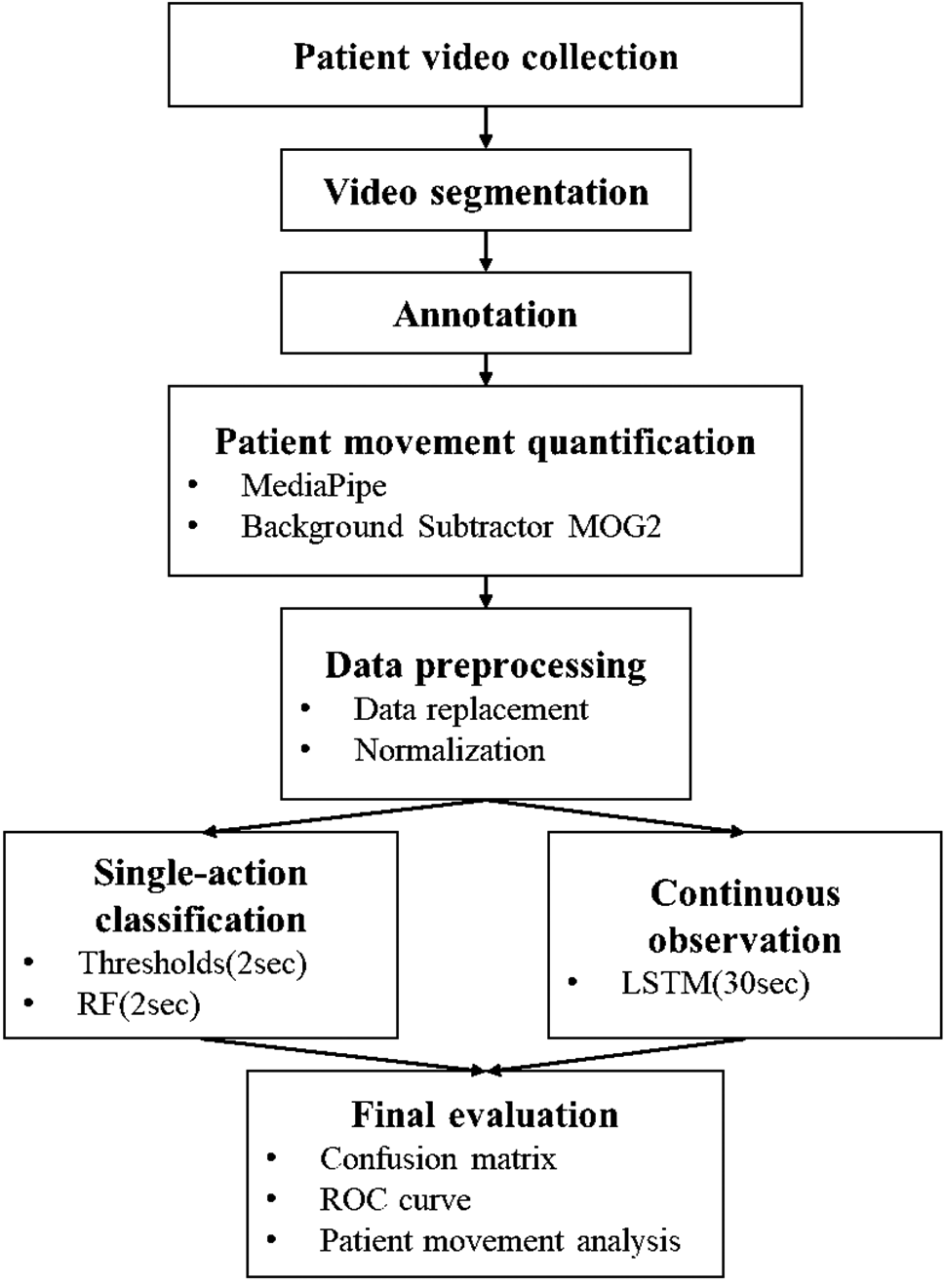
Research framework consisted of 7 steps: (1) patient video collection in ICU, (2) video segmentation, (3) annotation, (4) patient movement quantification (MediaPipe, Background Subtractor MOG2), (5) Data preprocessing (data replacement, normalization), (6) model construction with three methods, (7) evaluation of model performance

(1) patient video collection in the ICU, (2) video segmentation, (3) annotation, (4) patient movement quantification (MediaPipe, Background Subtractor MOG2), (5) Data preprocessing (data replacement, normalization), (6) model construction with three methods, (7) evaluation of model performance (Fig. 1).

#### Patient video collection

The digital video recordings were captured with a 4 K webcam at 1080p/30fps from the patients in the ICUs of Taichung Veterans General Hospital. Each patient, on average, had 8 min of recorded video footage.

#### Video segmentation

**Fig. 2:**
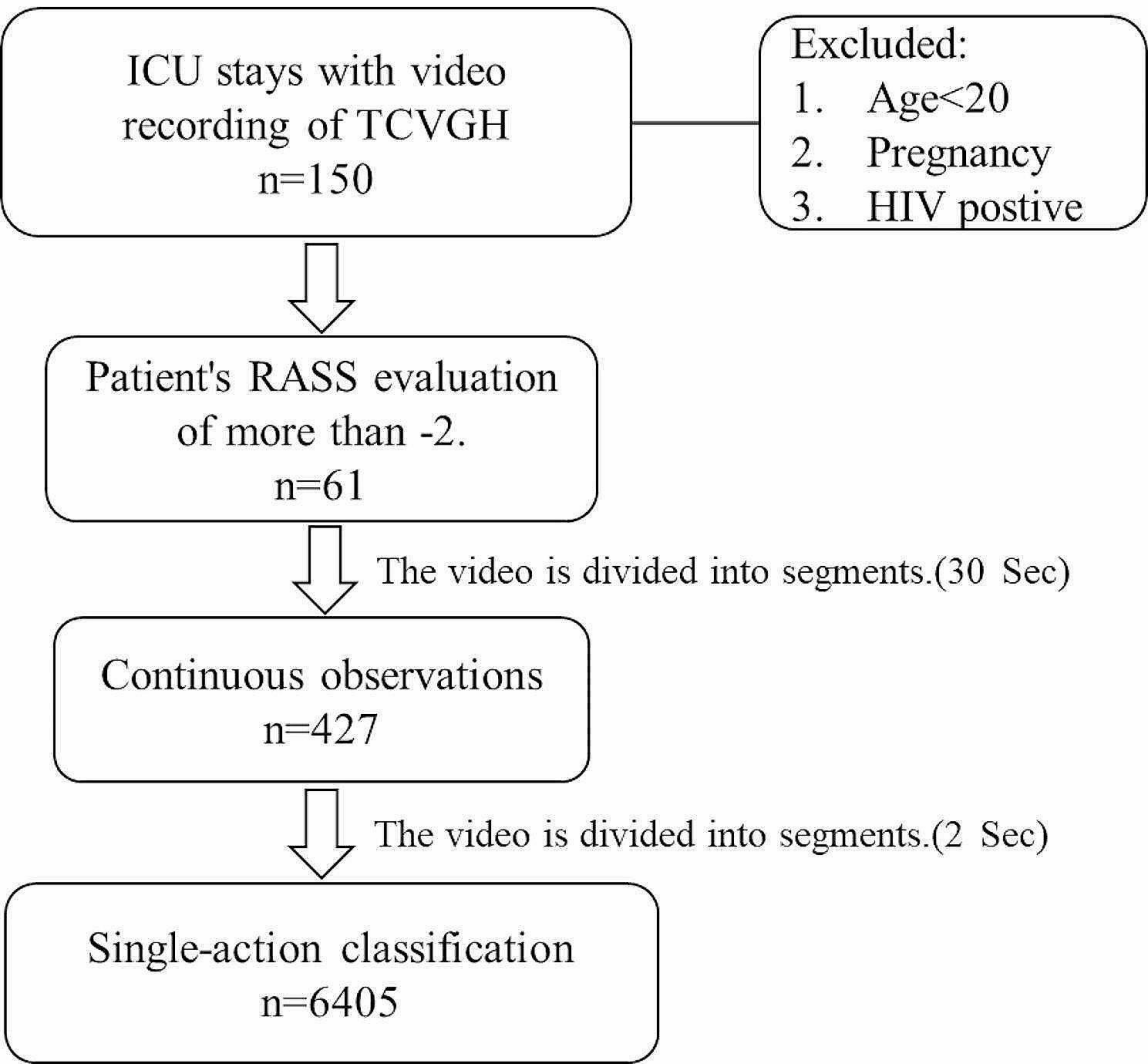
Data collection from patient enrollment to video recording segmentation

A subset of patients with the sedation level RASS ≤ -3 were excluded because of deep sedation and no movement. Finally, the study included 61 patients. Video recordings were cut into 30-second (30-s) intervals for continuous observation and categorization. Subsequently, each 30-s segment was cut into 2-second (2-s) sub-segments for single-action classification. Cases with more than 10 s of interference, such as caregiver interventions or camera shake within 30 s, were excluded. In total, 427 30-s segments for continuous observation and 6405 2-s sub-segments for single-action classification were obtained. (Fig. 2).

#### Annotation

Based on clinical experience, movements in different body regions pose varying levels of risk. For instance, raising the hands was considered high-risk, while lifting the feet was perceived as lower risk. This differentiation aids the model in learning movement patterns more precisely, ensuring a more accurate assessment of the patient’s condition.

Three experienced ICU nurses were invited for annotation. Before annotating, they discussed the agitated features of postures and movements (head, trunk, and lower limbs) (Table 1). They reached the consensus as the following. Ten cases were randomly selected and marked by two nurses based on patient activity. A third nurse assisted in consensus for cases with different annotations from the first and second nurses. They annotated another 20 cases to validate their consensus in classifying “attention” and “no attention”. Attention was defined as the video recordings showing patients resisting the belt restriction or moving limbs or heads out of the bed with agitation and safety risks, around equal to RASS 2 to 4. The others without the above conditions were labeled as “no attention”. They labeled all the 30-s and 2-s segments.

**Table 1:** Definitions for Body Regions in Evaluation Criteria

No	Project	Illustrate
1	Head	Using the shoulder joints on both sides as a benchmark, observe the areas where the head shakes or lifts.
2	Trunk (Include Upper limb)	Based on the shoulder and hip joints on both sides, observe the areas where the upper limbs are shaking or lifting.
3	Lower limb	Using the hip joints on both sides as a benchmark, observe the area where the lower limbs are shaking or lifting.
4	Physical Restraint	The process of using any implement, material, or device to immobilize the body in a manner that restricts an individual’s freedom of movement in their environment or approaches that individual’s physical freedom

#### Patient movement quantification

The MediaPipe machine learning framework developed by Google Research is highly valuable in the healthcare field. It is used to track hand movements and assess tremor in Parkinson’s disease, as well as diagnose low back pain by tracking joint positions in the body [14, 15]. Designed specifically for RGB video footage, the Pose model annotates 33 key joint positions for precise measurement.

**Fig. 3:**
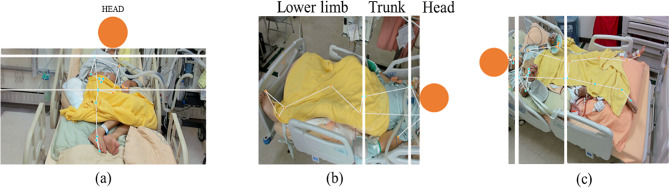
Patient’s posture determined by Using MediaPipe Pose in Various Cases: Vertical Position. Considering patient privacy, this paper presents only body parts outside the head, with the patient’s head represented by a circular symbol

This study determined the patient’s recumbent position (horizontal or vertical) by analyzing the distance between the y-coordinates of the left and right shoulders and between the right shoulder and right hip. For patients lying horizontally, the next step involved determining the head orientation by comparing the x-coordinates of the left shoulder node to the hip node. The head was above the coordinates of the right shoulder, the trunk was between the coordinates of the right shoulder and the right hip, and the lower limbs were below the coordinates of the right hip (Fig. 3).

**Fig. 4:**
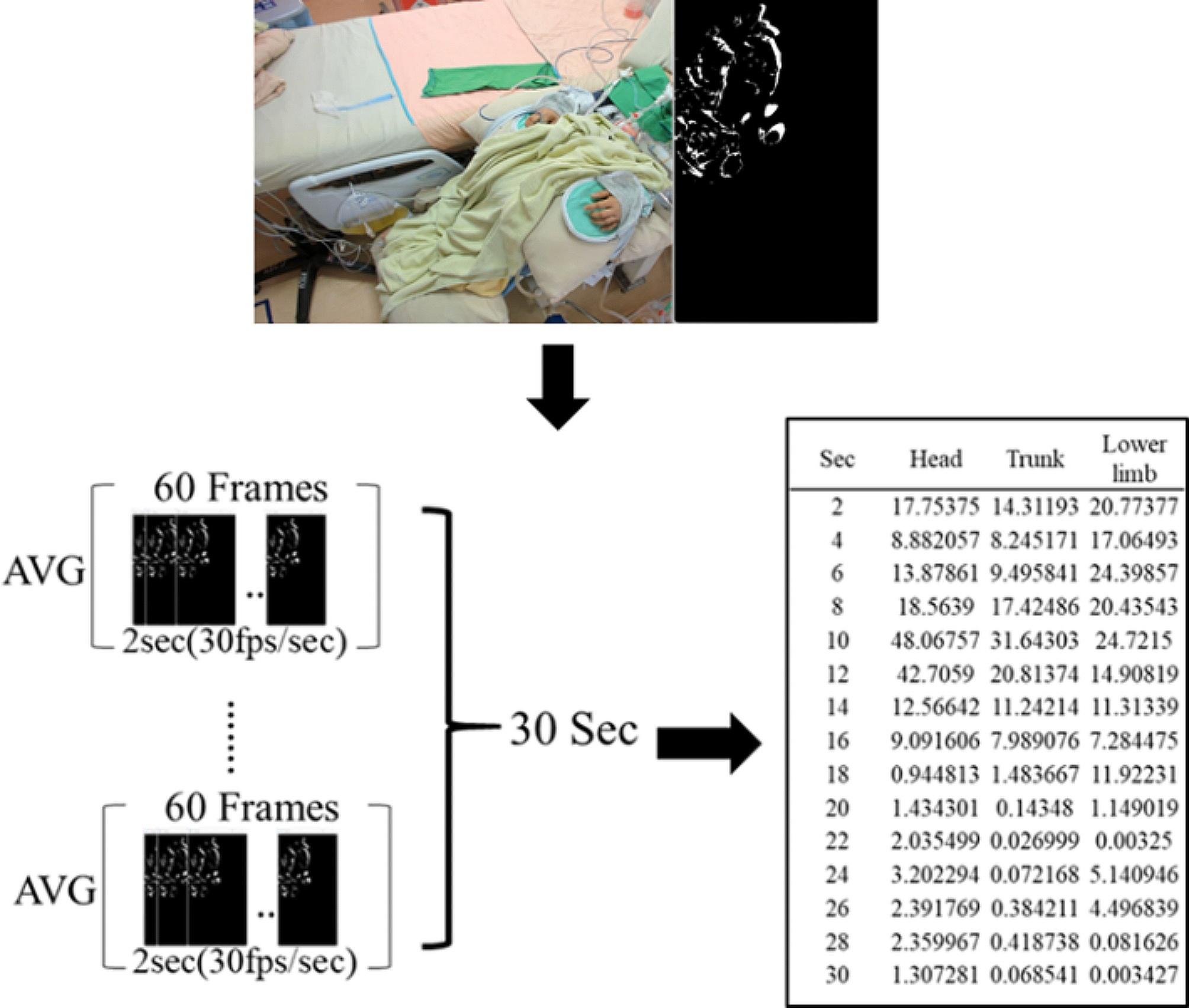
Background subtraction and patient motion quantification by OpenCV Background Subtractor MOG2 algorithm and Gaussian Mixture Models (GMM) [16]

The OpenCV Background SubtractorMOG2 algorithm utilizes Gaussian Mixture Models (GMM) for background separation in videos [16]. It learns the background and isolates moving foreground objects by associating each image pixel with a Gaussian distribution. The distribution weight reflects the duration of a color’s presence, helping identify the background. The algorithm effectively separates moving foreground objects. The process involves motion detection, converting the video into a black-and-white image. White areas indicate patient movement, and higher feature values represent more significant movement. These values are calculated by summing and averaging frames within each two-second interval (Fig. 4).

#### Data preprocessing

After converting the video into numerical data, this study replaced segments affected by external factors using the preceding adjacent numerical values to ensure that the model’s learning was not influenced. Additionally, the data is normalized to optimize the model parameters for this particular case.

#### Model construction

After preprocessing, the data was provided to the classification model. Thresholds and random forests were used for single-action classification (2-s).

1. Threshold:
  The threshold method classified the head, trunk, and lower limbs into three movement severity levels: no movement (1 point), bed movement (2 points), and significant off-bed movement (3 points). Scores for each body part’s classification results were aggregated (3 to 9 points). Thresholds for each body part and classification outcomes were determined using box plots, ensuring clinical requirements were met through confusion matrix indicators.
2. Random forests:
  The Random Forest (RF) algorithm, a highly effective classification method, excels in accuracy for big data scenarios [17]. Utilizing ensemble learning, RF constructs multiple decision trees during training, deriving predictions from identified patterns. This study applied RF to machine learning with quantized movement data, aiming to classify patients every two seconds. Key parameters were set as follows: n_estimators = 100, max_features = auto, criterion = Gini.
3. Long Short-Term Memor (LSTM):
  LSTM simulated continuous observations and classified patients every 30 s. The LSTM model used in this study, featuring 20 hidden units, 2 stacked layers, an input size of 4, and a time step of 15. In this study, model hyperparameters were fine-tuned, including data split ratios, activation function methods, and the consideration of data normalization. The training process was visualized, calculating losses and accuracies on the validation set after each epoch and recording metrics for both training and validation sets. Ultimately, a model with optimal stability and performance was chosen. The data was split into 80% training and 20% testing. We set the parameters for the validation, categorical cross-entropy loss function, adam optimizer, and softmax activation function. The validation data was from the 10% of training set.

#### Evaluation of model performance

In this study, confusion matrices and ROC curves are utilized as evaluation metrics, including accuracy (ACC), precision (P), recall (R), F1_Score, and cross-validation (kfold = 10) was applied to ensure model stability. The relationship between sensitivity and specificity is also depicted in the ROC curve, and the area under the ROC curve (AUC) value is calculated.

In addition to evaluating model performance, this study used line charts to analyze patient movement over 30 s. The goal was to confirm if the model’s assessments and image quantification align with real-world scenarios. Two representative cases have been selected. Cases of attention involved significant cross-zone movements, posing potential risks, while cases of no attention related to bed movements. Through motion analysis, the study clearly illustrated these distinctions and provided quantified results.

## Results

### System configuration

All experiments conducted in this paper were completed using the system configuration outlined in Table 2.

**Table 2:** System configuration

Environment	Python3.6
**Processor**	Intel Core i7 2.80 GHz
**Memory**	16GB
**Operating System**	Window 11

### Patients and video recording

We collected the video recordings of 150 patients. Only 61 patients with RASS scores ≥ -2 were enrolled for analysis. The average age was approximately 60 years old. Male patients predominated (M/F 46/15), particularly in videos featuring patients with a RASS score 0 (Table 3). The video recordings of the 61 patients were cut into 427 30-s and 6405 2-s segments (Fig. 2).

**Table 3:** Agitation status of the 61 patients

RASS scale	-2	-1	0	1	2	3	4
Total patients	14	6	36	1	4	0	0
Sex							
Male	10	5	26	1	4	0	0
Female	4	1	10	0	0	0	0
Age(avg)	63	59	67	40	65	0	0
Restraint	6	5	4	0	4	0	0

### Model construction

#### Thresholds definition

**Fig. 5:**
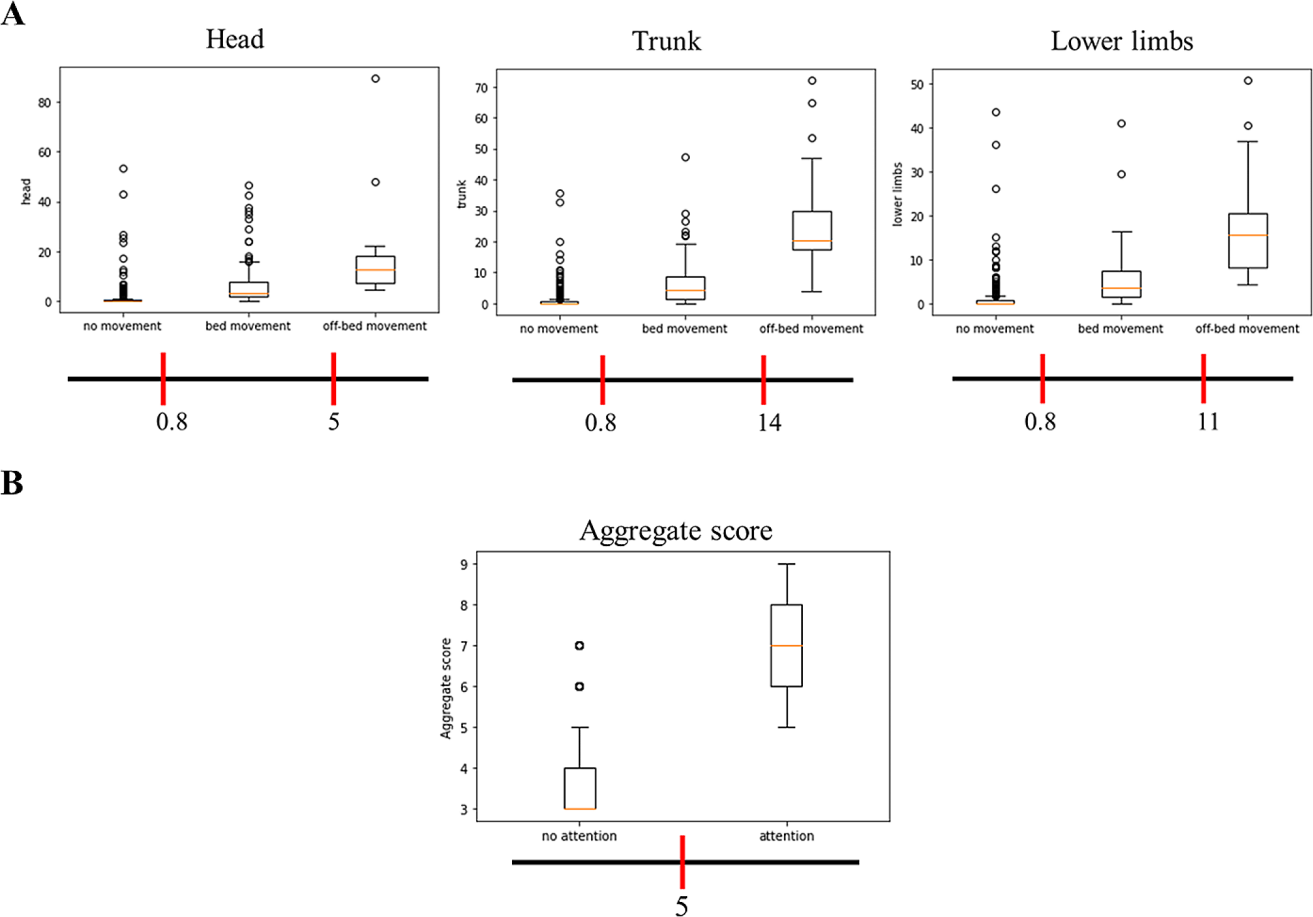
The threshold definitions by using a box plot. **(A)** Illustrates the threshold definitions for different body parts, with box plots encompassing quantified movement values and corresponding categories of no movement, in-bed movement, and significant out-of-bed movement. The bottom of the box plots presents the threshold definitions for various levels of movement. **(B)** Illustrates the threshold definitions for the aggregate score, with box plots comprising the aggregate scores for all body parts and their corresponding classification results (no attention and attention). The bottom of the box plots presents the threshold definitions for different categories

Figure 5 presents the threshold definitions by using a box plot. The specified cut-off values for different body parts were set at 0.8 and 5 for the head, 0.8 and 14 for the trunk, and 0.8 and 11 for the lower limbs (Figure 5A). Additionally, the aggregate scores for all body parts were subjected to a cut-off value of 5 (Figure 5B).

#### Model construction

In this study, the validation was conducted through confusion matrices and ROC curves to compare three classification methods. A cross-validation average accuracy(k-fold = 10) of RF and LSTM was 0.90. The LSTM model achieved the highest accuracy (ACC = 0.92). LSTM, using the time series data for classification, yielded the highest sensitivity (recall) for patients requiring attention and significantly improved various performance evaluation metrics (Table 4).

**Table 4:** Model performance among the models of Threshold, Random Forest, and LSTM

Method Classifications	Threshold (2-s)			Random Forest (2-s)			LSTM (30-s)		
	ACC:0.80			ACC:0.92			ACC:0.92		
	P	R	F1	P	R	F1	P	R	F1
Non-attention	1	0.78	0.88	0.95	0.96	0.96	0.96	0.92	0.94
Attention	0.34	0.98	0.50	0.67	0.60	0.63	**0.83**	**0.92**	**0.87**

**Fig. 6:**
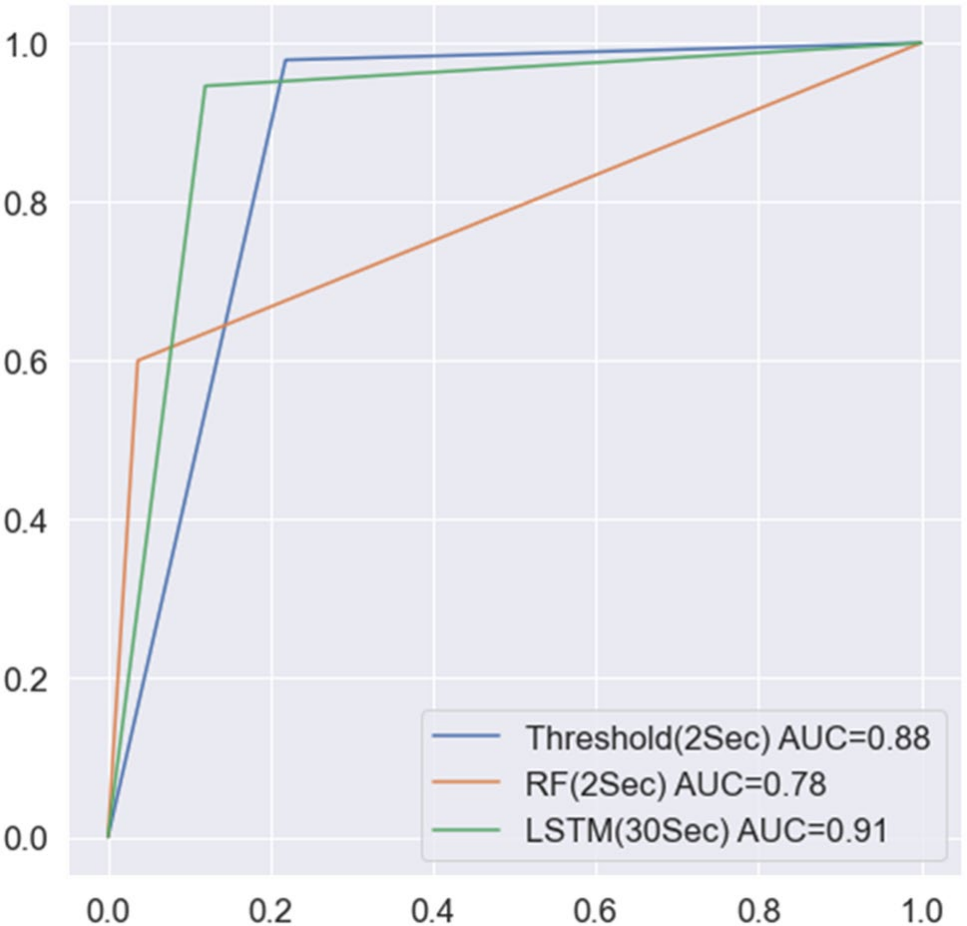
AUROC of Threshold, Random Forest, and LSTM. AUROC is the highest in the LSTM model

Additionally, by examining the ROC curve, it was found that the AUC performance of the LSTM model surpassed other methods (AUC = 0.91) (Fig. 6. This result emphasizes the outstanding performance of the LSTM model in simulating time series data of patient clinical observations.

### Patient movement analysis

#### Patient movement analysis of all case

We classified them into “Attention” and “Non-attention.“. We further stratified them into “Non-attention Without Restraint Belt,” “Non-attention With Restraint Belt,” “Attention Without Restraint Belt,” and “Attention With Restraint Belt.”

**Fig. 7:**
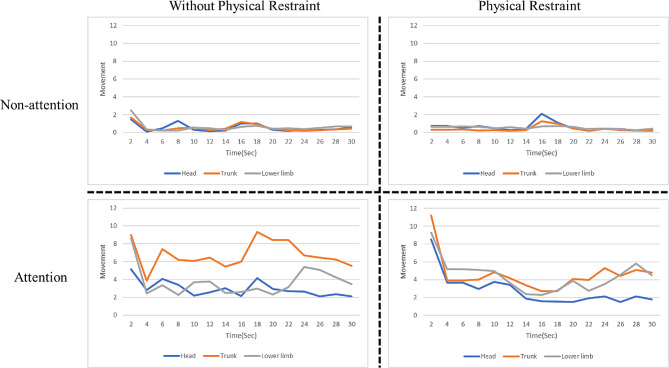
Patient movement analysis for All Cases: the x-axis illustrates motion quantified value, the y-axis denotes the time axis (seconds), and distinct colored lines represent different body parts

Referring to Fig. 7, it becomes apparent that the outcomes of image analysis align with clinical observations. There exists a notable contrast in movement between patients classified as “Attention” and those as “Non-attention.” Patients in the “Attention” category exhibit significantly more extensive movements, including those spanning different body regions. Within the “Attention” category, a noteworthy distinction surfaces between patients with and without restraint belts, with patients under restraint belts displaying reduced movement in the trunk area.

#### Patient movement analysis of the representative cases

**Fig. 8:**
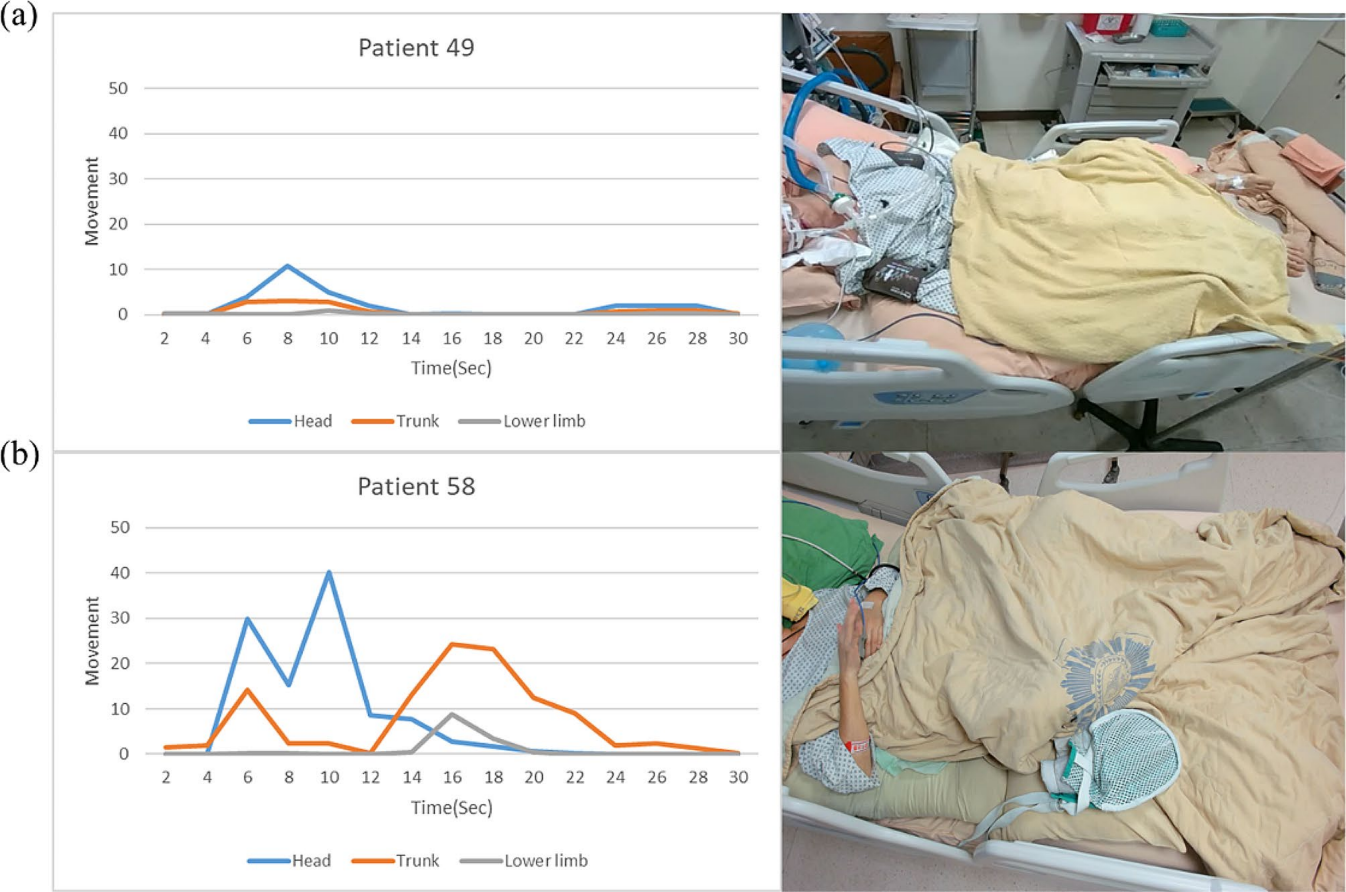
Patient movement analysis (left) and video(right)of individual cases. **(a)** Patient 49 **(b)** Patient 58

Patient 49 was categorized as “Non-attention,” with the image module detecting minimal head and limb movement. (Fig. 8a) Patient 58 was classified as “Attention,” with motion quantification revealing significant head and limb movements, including inter-regional motion (Fig. 8b). The analysis results from the image module align with the observed patient movements, demonstrating its accurate detection of displacement in each region. Due to privacy considerations, the patient’s head is not shown in the video. These movements are correlated with the analytical data, and corresponding videos will be included in the supplementary material.

## Discussion

Ensuring mild patient sedation in ICU care is crucial, but current clinical assessment methods encounter challenges like low frequency, subjectivity, and evolving professional standards, emphasizing the need for advanced, continuous monitoring methods [18, 19]. This study proposes a monitoring system that combines LSTM and image processing to address challenges such as ICU lighting variations and effective activity detection even when the patient is covered. The integrated AI technology enhances system accuracy, compensating for current monitoring limitations. Results align with expert observations.

Previous studies used cameras for agitation and sedation monitoring in the ICU. Chase et al. captured limb movements, quantifying sedation, and agitation levels using fuzzy logic methods [20]. Becouze et al. used cameras to record facial expressions, measuring agitation levels in critically ill patients [21]. Martinez et al. employed multiple cameras to observe patient behavior in the ICU for sedation control and accident prevention [22]. However, those researchers faced detection issues and a lack of detailed evaluation metrics.

This study compared three methods, and the results indicated that LSTM is the optimal choice. LSTM is renowned for its feature module’s selective retention of information and discarding unnecessary details, thereby having the potential to enhance performance [23]. Litton et al.‘s research demonstrated that expert-level diagnostic differentiation of various diseases can be achieved using electronic health records (EHR) and recurrent neural networks (RNN) [24]. LSTM technology aids healthcare professionals in diagnosis, prediction, and treatment, potentially enhancing efficiency and accuracy in the medical field and ultimately improving patient experiences and outcomes.

Despite significant progress, there is awareness of certain limitations. This study restricts the length and number of video segments related to patient safety and privacy concerns. These limitations include a relatively small number of cases and fewer cases with agitation (excluding 89 cases), along with marking only attention and no attention. However, practical judgment by clinical personnel confirms that this method holds clinical value in enhancing patient safety through continuous monitoring. Currently, manual interventions by healthcare professionals rely on manual pruning. Addressing these challenges requires improvements in smart device integration and workflows. Standardized methods, image transmission connections, and enhanced system security are crucial for monitoring system implementation and ensuring the legality, privacy, and reliability of the results. Future efforts can focus on expanding data collection, increasing the automation of medical interventions, and improving system integration and security to enhance practicality.

This study still holds significant value in clinical applications and provides solutions for future challenges. Despite existing challenges and risks, the potential benefits in patient care and reducing complications make these advancements promising for future clinical applications.

## Conclusion

Our study proposes an advanced monitoring system combining LSTM and image processing to address challenges in ICU care. It offers continuous and accurate monitoring, crucial for ensuring mild patient sedation amidst evolving standards and subjective assessments. LSTM emerges as the optimal choice, leveraging its information retention capabilities for enhanced performance, as seen in other medical applications.

While limitations exist due to patient safety and privacy concerns, our system holds clinical value in enhancing patient safety through continuous monitoring. Addressing these challenges requires improvements in device integration, workflows, and system security. Future efforts should focus on expanding data collection and enhancing system integration and security for practicality.

### Electronic supplementary material

Below is the link to the electronic supplementary material.

Supplementary Material 1

Supplementary Material 2

## Data Availability

All data supporting the findings of this study are available within the paper and its Supplementary Information.
